# Case report and literature review: removal of a mercury thermometer from the abdomen of a 16-year-old boy under laparoscopy

**DOI:** 10.3389/fsurg.2024.1465731

**Published:** 2024-12-10

**Authors:** Runjie Hou, Jijun Wang, Jing Guo, Mingyue Du, Zhenyu Dong, Xiaobiao Song

**Affiliations:** ^1^ Gastrointestinal Surgery Department, Baotou Central Hospital, Baotou, Inner Mongolia, China; ^2^ Baotou Medical College, Baotou, Inner Mongolia, China; ^3^ Department of Cardiothoracic and Vascular Surgery, Ordos Central Hospital, Ordos, Inner Mongolia, China

**Keywords:** abdominal cavity, foreign body, adolescent, laparoscopy, mercury thermometer, management strategy

## Abstract

**Introduction:**

The incidence of foreign bodies within the human body is uncommon, with thermometers representing an exceptionally rare subset of such cases. The management of these cases is particularly challenging due to the fragility of mercury thermometers and the toxic nature of their contents.

**Case description:**

A 16-year-old male adolescent presented with a three-month history of persistent, dull pain localized to the right inguinal region. Diagnostic imaging, including an abdominal upright x-ray and CT scans, revealed the presence of an intra-abdominal foreign body, specifically a thermometer. The diagnosis was subsequently confirmed intraoperatively through laparoscopic exploration. The foreign body was successfully extracted via laparoscopic intervention. The patient's postoperative course was uneventful, leading to discharge on the second day following the procedure. A one-month follow-up examination revealed no complications.

**Conclusion:**

A comprehensive literature review was conducted, focusing on cases involving thermometers as foreign bodies. The diagnostic and treatment experiences from the present case were integrated into this analysis. Based on these findings, a summary of diagnostic and treatment strategies for thermometer-related foreign body incidents has been formulated. It is recommended that an abdominal upright x-ray examination be employed as the primary diagnostic modality. The integrity and location of the thermometer, along with the presence of associated complications, should be considered as crucial factors in determining the most appropriate treatment approach. Furthermore, it is imperative to address the psychological and mental health aspects of these cases, particularly in adolescent patients.

## Introduction

Foreign bodies within the human organism present significant health risks, potentially leading to severe complications such as fistula formation, necrotizing pancreatitis due to direct organ perforation, vascular perforation-induced hemorrhage, and intestinal necrosis or obstruction resulting from luminal compression (1, 2). Mercury thermometers, owing to their fragile glass composition and the presence of toxic mercury, pose an even greater threat when broken, potentially causing more severe complications compared to other foreign objects. Prompt and accurate diagnosis, coupled with appropriate therapeutic intervention, is therefore of paramount importance in such cases. This study presents a successful case of laparoscopic extraction of a thermometer acting as a foreign body. A comprehensive review of pertinent literature has been conducted, and a summary of diagnostic and treatment strategies for thermometer-related foreign body incidents has been formulated.

## Case description

**Figure 1:**
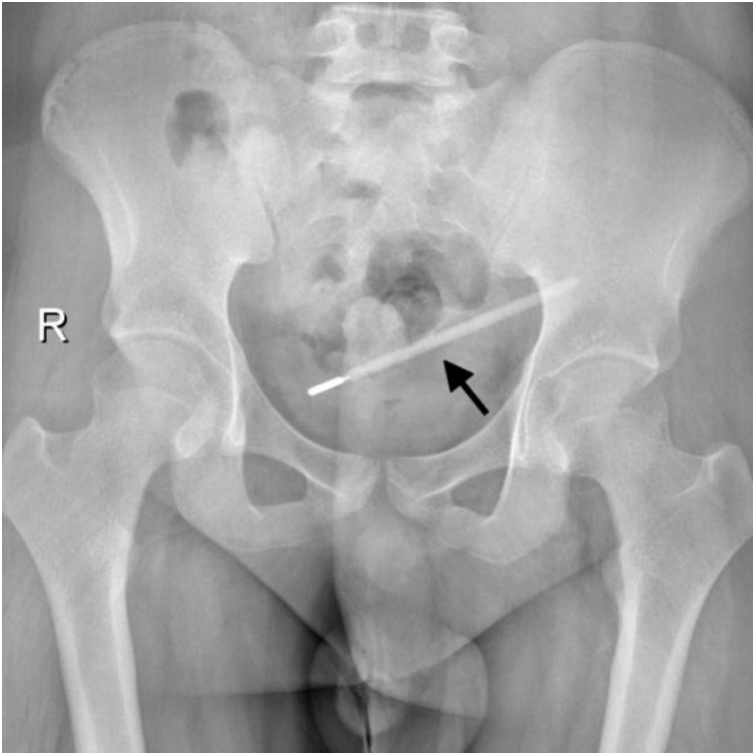
Abdominal upright x-ray showing a foreign body of a thermometer (the site pointed to by the arrow).

**Figure 2:**
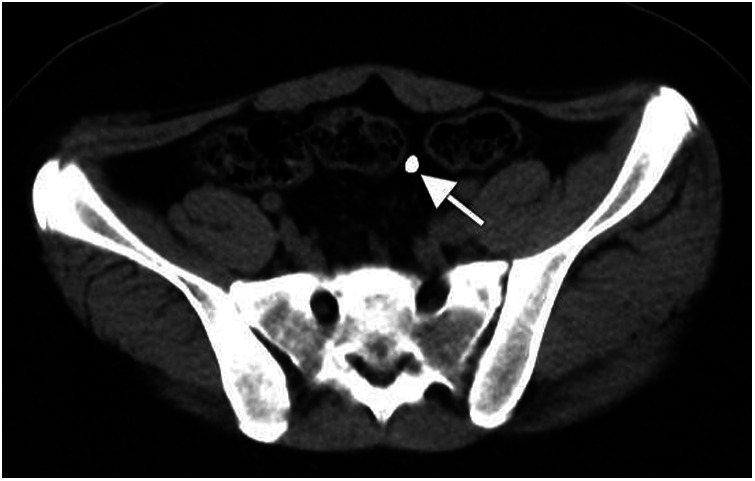
Ct scan showing intraperitoneal localization of the thermometer (the site pointed to by the arrow).

**Figure 3:**
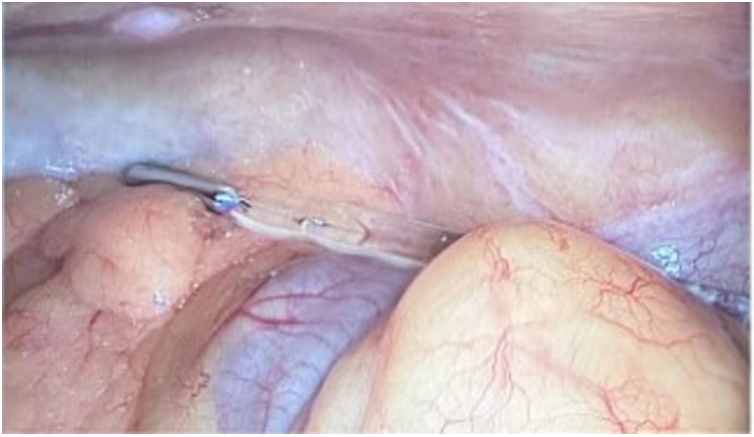
Exploration revealed an intact and free thermometer in the abdominal cavity.

**Figure 4:**
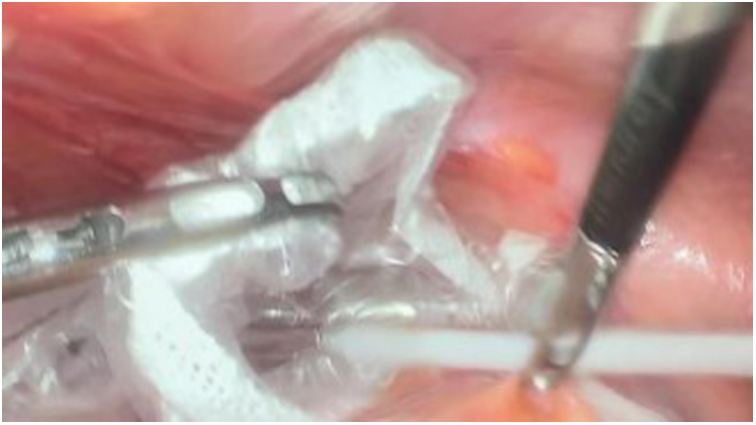
Placing the thermometer in the self-made specimen bag.

**Figure 5:**
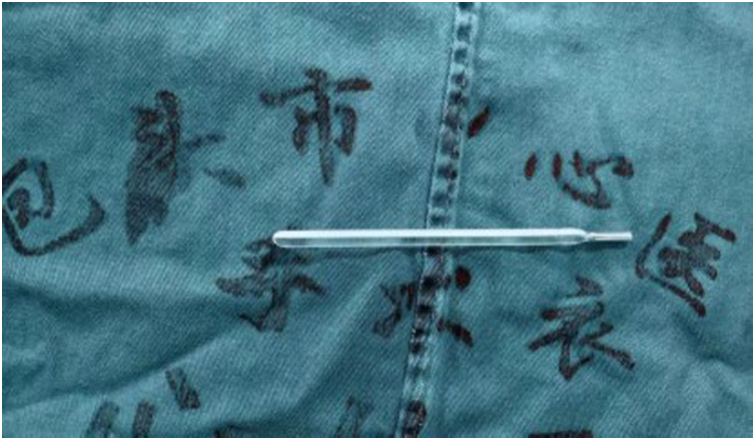
Removal of the thermometer.

A 16-year-old male adolescent presented to the gastrointestinal surgery department with a three-month history of dull pain localized to the right inguinal region. Physical examination in the supine position revealed a soft abdomen without tenderness. An abdominal upright x-ray was performed, revealing a high-density foreign body shadow in the lower abdomen, consistent with the appearance of a thermometer (Figure 1). Upon detailed medical history taking, the patient admitted to inserting a mercury thermometer through the urethra three months prior, initially refraining from seeking medical attention due to embarrassment. An abdominal computed tomography (CT) scan was subsequently conducted to precisely localize the thermometer (Figure 2). Based on these findings, a decision was made to proceed with laparoscopic exploration for removal of the foreign body. Following preoperative preparation, the patient underwent general anesthesia and laparoscopic exploration the following day. Upon entering the abdominal cavity, no active bleeding, purulent exudate, or effusion was observed. An intact mercury thermometer was identified in the right iliac fossa, floating freely within the abdominal cavity (Figure 3). A self-made specimen bag was utilized to enclose the thermometer (Figure 4), which was subsequently extracted through the left trocar port (Figure 5). A thorough examination of the abdominal organs revealed an old scar at the base of the bladder, while the remaining organs appeared intact. Bladder integrity was confirmed by instilling 200 milliliters of saline through a catheter, with no observed fluid leakage. The procedure was deemed successful, and the patient was discharged the following day.

## Literature review

A comprehensive literature search was conducted using the PubMed/MEDLINE database, focusing on case reports and original studies pertaining to intra-abdominal thermometer foreign bodies. The search encompassed publications from January 1, 2014, to June 28, 2024, without language restrictions. The search strategy employed the combination of terms [(thermometer) and (foreign body)]. This systematic approach yielded nine relevant articles, collectively reporting ten cases of thermometer foreign bodies. One article described two distinct cases of thermometer foreign bodies (Table 1).

**Table 1:** Studies on cases of foreign bodies related to thermometers throughout the literature.

Report authors, years	Thermometer types	Country	Sex	Age at injury (years)	Entry site	Position (at the time of thermometer removal)	Integrity (thermometer)	Symptoms	Time interval between injury and surgery	Diagnostic method	Intervention required	Organs damage
Chengpin Tao (2024) (3)	Mercury thermometer	China	Male	13	Transurethral insertion	Urethra	Intact	Urinary tract infection	Unknown	Ultrasonography	Open surgical removal	None
Chengpin Tao (2024) (3)	Mercury thermometer	China	Male	12	Transurethral insertion	Urethra	Intact	Dysuria	Unknown	x-ray	Open surgical removal	None
Katrin Schulte (2024) (4)	Not mentioned	Switzerland	Male	67	Oral ingestion (a total of 12 thermometers)	Urethra, scrotum, duodenal, gastric, abdominal cavity, and terminal ileum	Intact	Abdominal pain	40 years	x-ray, CT	Open surgical removal	Duodeno-sigmoid fistula, necrosis of the right hemicolon, retroperitoneal abscess, and gastro-thoracic fistula
Changyi Jiang (2023) (5)	Mercury thermometer	China	Male	12	Transurethral insertion	Bladder	Intact	Lower abdominal pain	9 h	x-ray	Endoscopic removal	None
Liu Yang (2021) (6)	Mercury thermometer	China	Male	32	Oral ingestion	Neck (extra-esophageal)	Incomplete (pre-swallow)	Restricted neck movement	5 years	x-ray, CT	Open surgical removal	None
Ganggang Yang (2019) (7)	Mercury thermometer	China	Male	25	Transurethral insertion	Bladder	Intact	No discomfort	11 days	x-ray	Endoscopic removal	None
Jovo Bogdanović (2017) (8)	Mercury thermometer	Serbia	Male	63	Transurethral insertion	Intraperitoneal (via bladder perforation migration)	Intact	No discomfort	10 h	x-ray, CT	Laparoscopic removal	Bladder perforation
Vassilis Lambropoulos (2016) (9)	Mercury thermometer	Malaysia	Female	6	Oral ingestion	Appendix	Incomplete (pre-swallow)	No discomfort	2 months	x-ray	Open surgical removal (appendicectomy)	None
Jing Nie (2014) (10)	Not mentioned	China	Male	45	Transurethral insertion	Intraperitoneal (via bladder perforation migration)	Intact	Intestinal obstruction	2 years	x-ray, CT	Open surgical removal	Bladder perforation, intestinal obstruction
M. Dardamanis (2014) (11)	Not mentioned	Greece	Female	48	Transurethral insertion	Bladder	Intact	Renal injury	3 months	x-ray	Open surgical removal	Acute obstructive renal injury; 5 cm bladder stone

## Discussion

Intra-abdominal foreign bodies are relatively uncommon occurrences. These foreign bodies can be classified into two categories based on their route of entry: (1) those that migrate into the abdominal cavity through perforation of natural hollow organs, such as the gastrointestinal, urinary, and reproductive tracts, and (2) those that enter directly through breaches in the skin (12). The presence of foreign bodies within the human organism poses significant health risks, potentially leading to severe complications. These complications include fistula formation, necrotizing pancreatitis due to direct organ perforation, hemorrhage resulting from vascular perforation, and intestinal necrosis or obstruction caused by compression and blockage of the intestinal lumen (1, 2). Prompt intervention is crucial upon the discovery of a foreign body to mitigate these risks.

The presence of thermometers as foreign bodies was initially documented in the mid-20th century, coinciding with the advocacy of vaginal temperature measurement for ovulation date estimation (13). In recent years, despite the gradual replacement of mercury thermometers with gallium alternatives due to environmental and toxicity concerns (14), mercury thermometers remain in use in many regions owing to their cost-effectiveness and superior accuracy. Consequently, cases of mercury thermometers as foreign bodies continue to be reported. Recent literature predominantly describes male patients inserting thermometers into the urethra for psychiatric reasons or sexual gratification, with a notable trend towards younger patients (Table 1). Thermometers, particularly those containing mercury, present unique challenges as foreign bodies compared to other objects. The fragility of their glass construction and the presence of mercury pose dual risks. While the potential for severe injury from broken glass is significant (14), it is noteworthy that based on previous reports and the findings of this case study, accidental intracorporeal thermometers have not been observed to rupture spontaneously, even after prolonged periods of up to 40 years within the body (4). This may be attributed to the thermometer's smooth shape, the protective nature of internal organs, and the lubrication provided by bodily fluids. However, the sharp configuration of thermometers renders them more susceptible to puncturing internal organs, potentially leading to fistula formation and perforation (3). The leakage of mercury from a ruptured thermometer presents an additional hazard. While the absorption rate of mercury through a healthy gastrointestinal tract is less than 0.01%, rendering mercury poisoning unlikely in cases of thermometer rupture within the gastrointestinal tract (15), the consequences of rupture within the abdominal cavity following perforation of a hollow organ would be severe. In such instances, peritoneal absorption of mercury could lead to central nervous system damage, manifesting as insomnia, fatigue, and memory loss, as well as peripheral nerve and kidney damage (16, 17). Furthermore, chronic inflammation resulting from mercury exposure could lead to the formation of difficult-to-heal fistulas, the development of granulomas, and intestinal obstruction (17).

The clinical manifestations resulting from the presence of a thermometer as a foreign body within the human organism are typically subtle, with the majority of patients remaining asymptomatic. This lack of apparent symptoms may be attributed to the thermometer's smooth surface, which minimizes irritation to internal organs. A comprehensive medical history, when obtainable, significantly enhances the formulation of diagnostic and treatment strategies. However, factors such as patient embarrassment and underlying mental health issues (3) often impede the acquisition of an accurate history of thermometer insertion. For instance, the patient in the present case initially exhibited reluctance to disclose the self-insertion of the thermometer. Nevertheless, the diagnosis of a thermometer as a foreign body can be established with relative ease, even in the absence of a detailed ingestion history. Based on previous case reports and the current study, it is evident that intra-abdominal thermometers can be readily identified on radiographic images due to their radiopaque nature and distinctive morphology. This characteristic allows for a preliminary diagnosis through a simple x-ray examination. Further evaluation of the spatial relationship between the thermometer and surrounding organs, as well as any associated injuries, can be achieved through CT scans.

**Figure 6:**
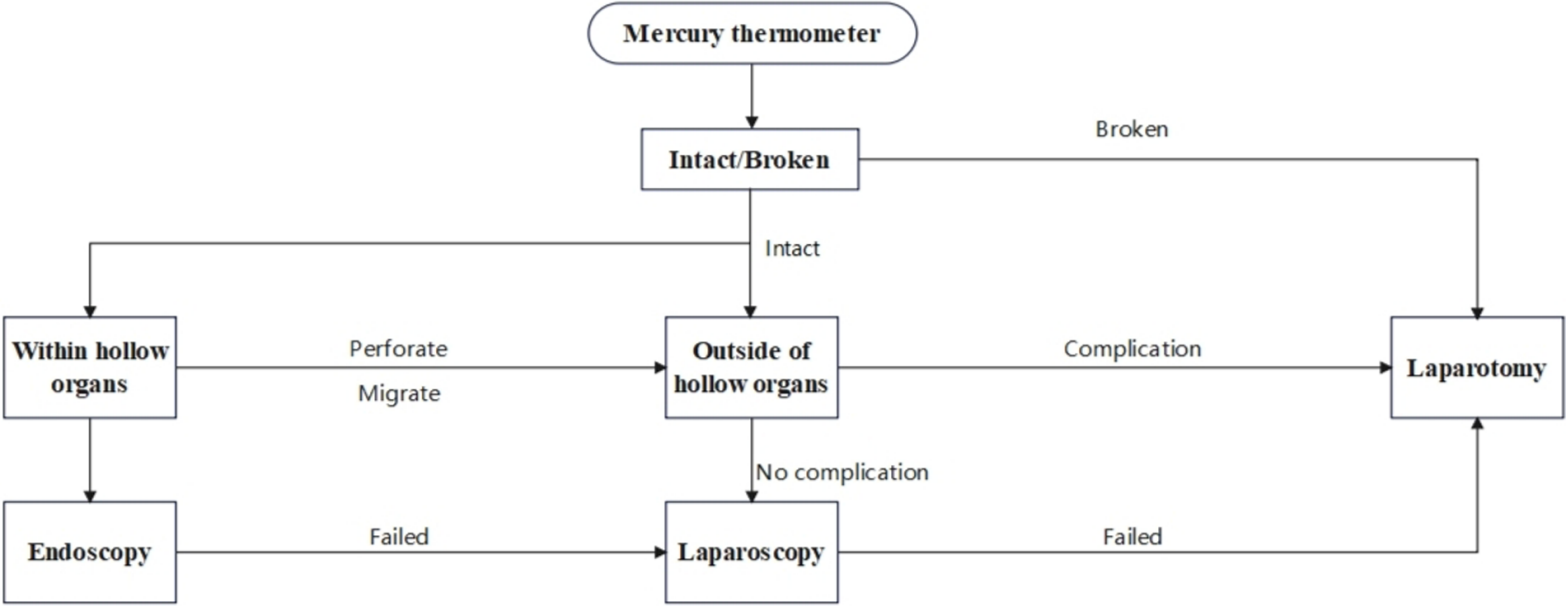
Management of mercury thermometer foreign bodies,recommended by the authors.

Drawing from previous reports and the present case, a summary of treatment modalities for thermometer foreign bodies has been compiled (Figure 6). The initial step involves determining the integrity of the thermometer. In cases where the thermometer is found to be broken, immediate open surgery is warranted, with potential removal of contaminated organs if necessary. For intact thermometers that have not penetrated the bladder, uterus, digestive tract, or other hollow organs, endoscopic intervention is the preferred approach (5, 7). When the thermometer has penetrated hollow organs and entered the abdominal cavity, laparoscopic exploration and removal are favored to minimize surgical trauma. To mitigate the risk of thermometer breakage and its consequent serious complications during laparoscopic exploration, a protective cover can be fabricated to encase the thermometer prior to extraction. In scenarios where the thermometer is found to be firmly adherent to organs and difficult to separate, partially embedded in the intestinal tract or bladder, or has caused complications such as perforation or obstruction, prompt open surgery should be performed instead of attempting laparoscopic removal to avoid exacerbating the damage (10). Furthermore, addressing the patient's mental health is of paramount importance. Patients presenting with bladder and urethral foreign bodies, particularly male patients, often exhibit concomitant psychiatric disorders (18, 19), necessitating a comprehensive evaluation of their mental state. Adolescent patients, who are often driven by sexual curiosity (5), require appropriate psychological counseling to prevent the recurrence of foreign body insertion. This multifaceted approach ensures not only the physical removal of the foreign body but also addresses the underlying psychological factors that may have contributed to the incident.

## Conclusion

A systematic strategy for the diagnosis and management of thermometer foreign bodies has been formulated based on a comprehensive review of relevant literature from the past decade, coupled with the clinical insights gained from the present case study. Abdominal upright x-ray examination is recommended as the primary diagnostic modality. The integrity and location of the thermometer, along with the presence of any associated complications, are identified as critical factors influencing the selection of appropriate treatment strategies. Furthermore, significant emphasis is placed on the psychological and mental health status of patients, particularly adolescents, necessitating the provision of tailored psychological counseling and therapeutic interventions.
